# Inhalable Jojoba Oil Dry Nanoemulsion Powders for the Treatment of Lipopolysaccharide- or H_2_O_2_-Induced Acute Lung Injury

**DOI:** 10.3390/pharmaceutics13040486

**Published:** 2021-04-02

**Authors:** Guoli Zhang, Fei Xie, Yunbo Sun, Xiang Yu, Zhimei Xiao, Rongzhen Fang, Jingfei Li, Qian Li, Lina Du, Yiguang Jin

**Affiliations:** 1 Graduates Department, Anhui Medical University, Hefei 230032, China; guolizhang66@163.com; 2 Department of Pharmaceutical Sciences, Beijing Institute of Radiation Medicine, 27 Taiping Road, Beijing 100850, China; sunyunbo0919@126.com (Y.S.); 17858779668@163.com (X.Y.); xiaozhimei18950@163.com (Z.X.); fang805045277@163.com (R.F.); ljf13100960516@163.com (J.L.); 17862969577@163.com (Q.L.); 3 Department of Pulmonary and Critical Care Medicine, Chinese PLA General Hospital, Beijing 100853, China; xiefei0522@163.com; 4 Pharmaceutical College, Henan University, Kaifeng 475004, China; 5 School of Medicine, Shandong University of Traditional Chinese Medicine, Jinan 250355, China

**Keywords:** jojoba oil, acute lung injury, nanoemulsion, dry powder inhaler, hydrogen peroxide, lipopolysaccharide (LPS)

## Abstract

Jojoba (Simmondsia chinensis (Link) C.K. Schneid) is a dioecious plant in desert and semi-desert areas, e.g., the Ismailia Desert in Egypt. Jojoba oil (JJBO) is a natural slight yellow oil with the functions of skin barrier repairing and wound healing, which is dermally applied as a traditional medication or cosmetic in the Middle East. The objective of this study was to prepare JJBO dry nanoemulsion powders (JNDs) and investigate their anti-acute lung injury effects. JJBO nanoemulsions (JNEs) were prepared and then lyophilized to JNDs and the properties and simulated lung deposition were measured. Rat acute lung injury (ALI) models were established after intratracheal (i.t.) administration of lipopolysaccharide (LPS) or hydrogen peroxide (H_2_O_2_). JNDs and dexamethasone (DXM) solutions were also i.t. administered to the rats. The pathological states of lung tissues were checked. Inflammatory and oxidative factors in the lung tissues were determined using ELISA methods. NF-κB p65 and caspase-3 were measured with a Western blotting method and an immunohistochemical method, respectively. JNDs had an appropriate mass median aerodynamic diameter (MMAD) of 4.17 µm and a fine particle fraction (FPF) of 39.11%. JNDs showed higher anti-inflammatory effect on LPS-induced ALI than DXM with a decrease in total protein content and down-regulation of tumor necrosis factor-α (TNF-α), interleukin-1β (IL-1β), interleukin-6 (IL-6) and NF-κB p65. JNDs also showed higher anti-inflammatory and anti-oxidation effect on H_2_O_2_-induced ALI than DXM with elimination of reactive oxygen species (ROS), increasing of superoxide dismutase (SOD), decrease in of lipid peroxide malondialdehyde (MDA) and glutathione (GSH), and inhibition of caspase-3 expression. Moreover, i.t. JNDs attenuated bleeding and infiltrations of the inflammatory cells in the two ALI models. JNDs are a promising natural oil-contained inhalable medication for the treatment of LPS- or H_2_O_2_-induced ALI.

## 1. Introduction

Jojoba (Simmondsia chinensis (Link) C.K. Schneid) is a dioecious plant in desert and semi-desert areas including the Ismailia Desert in Egypt [1,2]. Jojoba oil (JJBO) is a slightly yellow liquid. JJBO contains 97% linear long-chain esters and other components including polyphenols, flavonoids, and alkaloids [3,4]. JJBO is used in folk remedies for renal colics, headache, sunburn, chaffed skin, and hair loss [5]. The traditional medicinal applications of JJBO are mainly limited to skin care, involving skin barrier repairing [6], and wound healing [5]. Dermatological research shows that JJBO can reduce inflammation for the treatment of acne and psoriasis [7]. Moreover, JJBO shows high safety without significant toxicity and side effects [8].

Acute lung injury (ALI) is a series of syndromes characterized by the infiltration of inflammatory cells, edema of the lung, and gas exchange disorders. ALI is mainly caused by excessively uncontrolled inflammatory reactions and the imbalance of pro-inflammatory and anti-inflammatory responses. ALI is generally the result of pathological diffusion of pulmonary capillary membranes and alveolar damages due to the severe infection of viruses [9], trauma, inhalation of toxic gases, shock, poisoning, and other injuries [10]. Acute respiratory distress syndrome (ARDS) is an extremely serious state or the terminal stage of ALI with high mortality of 35–40% [11]. Lipopolysaccharide (LPS) is one major components of the cell wall of Gram-negative bacteria, which may be released into the surroundings as the bacteria are dead. The accumulation of LPS in the lung may induce a strong local immune response, resulting in ALI [12]. Now air pollution becomes a serious social problem in many developing countries. Inhalation of smoke, dust and toxic gases would likely produce a great number of oxidative free radicals in the lung, leading to damages of pulmonary vascular endothelial cells/alveolar epithelial cells to form ALI [13].

There is no specific medication for the treatment of ALI. Only some systemic therapies, such as glucocorticoids [14], are clinically available. However, short-term therapy is usually not satisfied while some severe side effects would likely happen with long-term therapy. Pulmonary drug delivery is an optimal method for the treatment of lung diseases due to high drug lung distribution, and it is regarded by the World Health Organization as the first choice for respiratory diseases, especially for the rapid treatment of acute lung diseases [15,16]. Inhalation of glucocorticoids is clinically used [17], though some side effects still happen after a large dose or a long-term therapy is given. A new coronavirus, called SARS-CoV-2019, has swept throughout the world from the end of 2019 [18]. Half of the patients develop dyspnea after infection for one week and quickly progress to ARDS due to severe ALI. Therefore, a safe and effective medication is needed for the treatment of ALI.

Here, we prepared the inhalable formulations of JJBO for the treatment of LPS- or H_2_O_2_-induced ALI. The preparation of oil-based inhalers is a challenge [19]. JJBO nanoemulsions were prepared and transformed into inhalable dry powders. The optimal formulation and preparation process of nanoemulsions and inhalable powders were explored. The anti-ALI therapeutic effect of intratracheal (i.t.) JJBO powders were investigated on ALI rats and the mechanisms were analyzed. Moreover, for the first time, we found that JJBO and its formulations had an anti-free radical function. To our knowledge, this is the first research on inhaled active natural oils for the treatment of ALI after transformation from oil liquids to inhalable fine powders.

## 2. Materials and Methods

### 2.1. Materials

Jojoba oil was provided by Yangzhou Joyvo WeiKem Biological Co., Ltd., (Yangzhou, China) and identified by Pure Life Company for Agricultural Investment and Development (Giza Egypt). The voucher specimen (No. 20190311) was kept in the group’s laboratory. Egg yolk lecithin (PL-100M, injection grade) was purchased from A.V.T. (Shanghai) Pharmaceutical Co., Ltd., Beijing, China. Lactose (SuperTab 14SD) was purchased from DMW-Fonterra Excipients Gmbh & Co. KG., Goch, Germany. Mannitol and hydrogen peroxide (H_2_O_2_) were purchased from Sinopharm Chemical Reagent Co., Ltd., Shanghai, China. Dexamethasone Sodium Phosphate Injection was purchased from Ruicheng Tiantong Dongbao Co., Ltd., Beijing, China. Lipopolysaccharide (LPS) was purchased from Sigma, USA. Rat TNF-α, IL-1β and IL-6 ELISA Kits were purchased from Neobioscience Technology Co., Ltd., Beijing, China. The kits of malondialdehyde (MDA), superoxide dismutase (SOD), and micro-reduced glutathione (GSH) tests were purchased from Nanjing Jiancheng Bioengineering Institute, China. A Rat ROS ELISA kit was purchased from Shanghai Lianmai Biotech Co., Ltd., Shanghai, China. A bicinchoninic acid (BCA) protein concentration test kit was purchased from Beijing Solarbio Science & Technology Co., Ltd., Beijing, China. A total polyphenol kit was purchased from Shanghai Yi-tsung Industrial Co., Shanghai, China. 1,1-diphenyl-2-picrylhydrazyl (DPPH) was purchased from Beijing InnoChem Science & Technology Co., Ltd., Beijing, China. Purified water was prepared using the Heal Force Super NW Water System (Shanghai Canrex Analytic Instrument Co., Ltd., Shanghai, China). All other chemicals and solvents were of analytical grade.

### 2.2. Animals

Male Sprague Dawley (SD) rats (180–220 g), purchased from Beijing Vital River Experimental Animal Technology Co. Ltd., Beijing, China (Certificate number: SCXK (JING) 2016-0006), were used. The handling and surgical process of animals were strictly performed according to the Guiding Principles for the Use of Laboratory Animals of the Beijing Institute of Radiation Medicine. The experiment was approved by the Committee of Experimental Animal Ethics of the Beijing Institute of Radiation Medicine (No. IACUC-DWZX-2020-534).

### 2.3. Preparation and Characterization of JJBO Nanoemulsions

Egg yolk lecithin (0.4 g) was dissolved in ethanol (5.5 mL) followed by the addition of JJBO (0.55 g). Purified water (73.5 mL) was dropped into the above solution that was then homogenized at 30,000 rpm using a high-speed disperser (D-1, MICCRA GmbH, Heitersheim, Germany) until to obtain homogenous white emulsions, i.e., JJBO nanoemulsions (containing 0.69% JJBO, w/w).

The particle sizes, size distribution and zeta potentials of JNEs were measured with the dynamic light scattering method on a Zetasizer Nano ZS (Malvern, UK) at 25 °C. The morphology of JNEs was observed under a Hitachi H-7650 80-kV transmission electron microscope (TEM) after they were stained with a 2% sodium phosphotungstate solution (pH 7.2).

### 2.4. Preparation and Characterization of JJBO Nanoemulsion Dry Powders

JJBO nanoemulsion dry powders (JNDs) were prepared by lyophilization of JNEs that were mixed with 3.75% lactose. The nanoemulsions were freeze-dried as soon as possible in a lyophilizer (LGJ-30F, Beijing Songyuan Huaxing Technology Develop Co., Ltd., Beijing, China) referring to the previous process [20]. JNDs were sieved through 180-mesh sieves. The bulk density and tap density of the fine powders were measured with the cylinder method (*n* = 3). Median geometric diameter (*D_e_*) was measured with a particle size analyzer (BT2001, Bettersize Instruments Ltd., Dandong, China) and the median aerodynamic diameter (*D_a_*) was calculated as Equation (1).

(1) $$D_{a}=D_{e}\sqrt{\frac{\rho_{p}}{\rho_{0}\chi }}$$

where *ρ_p_* is the effective particle density that is 1.26 times the tap density; *ρ*_0_ is the reference density, equal to 1 g/cm^3^; *χ* is the dynamic morphological factor (*χ* = 1 when spherical, 1 used in this paper) [21]. JNDs were reconstituted with water (about 10:1, water/JNDs, w/w) followed by observation under TEM. Moreover, JNDs were adhered to conductive tapes and coated with gold powders followed by observation under a scanning electron microscope (SEM, S4800, 10 kV, Hitachi, Tokyo, Japan).

### 2.5. Simulated Lung Deposition of JNDs

Simulated lung deposition of JNDs was investigated using Next Generation Impactor (NGI, Copley, Nottingham, UK). JNDs were filled into hydroxypropyl methylcellulose (HPMC) hard capsules (Capsugel, Type 3, Suzhou Capsule Ltd., Suzhou, China) with 20 mg each capsule. A linked pump was actuated to simulate an inspiration (air flow rate of 60 L/min for the duration of 7 s). Twenty capsules of JNDs were used. The particles deposited on the capsule, inhaler, adapter, throat, Stages 1–7 and micro-orifice collectors (MOC) were collected using ethanol, respectively. The polyphenols in every sample were determined using the method in Section 2.6. The fine particle fraction (FPF) of JNDs was calculated as Equation (2) [22].

FPF = (M_3–7_)/(M_total_) × 100% (2)

where M_3–7_ was the amount of JJBO deposited in the Stage 3-7 collectors, and M_total_ was the total amount of JJBO in all the compartments. The mass median aerodynamic diameter (MMAD) and geometric standard deviation (GSD) of JNDs were calculated using NGI software (Copley Inhaler Testing Data Analysis Software, Version 3.10 Wibu USP32/Ph. Eur. 6.0, the COPLEY company, Nottingham, UK).

### 2.6. Measurement of Polyphenols in JJBO Formulations

Polyphenols are the major ingredients of JJBO [23]. They can be measured using the polyphenol content kit [24]. JJBOs were completely dissolved in absolute ethanol to form a transparent solution, where polyphenols were determined with the polyphenol content kit and a Purkinje TU-1901 spectrophotometer (Beijing Purkinje General Instrument Co., Ltd., Beijing, China) at 765 nm. JNDs were thoroughly mixed with absolute ethanol and the polyphenols in the supernatant were measured as above.

### 2.7. Pharmacodynamics Study In Vivo

LPS-induced ALI was selected in this study, which is the representative of biological ALI [7,25]. Rats were fixed with hands. A laryngoscope was used to visualize the trachea opening. LPS (0.2 mL, 5 mg/mL) was intratracheally (i.t.) administered to the rat via a soft plastic tube. One hour later, medications were also i.t. administered via a soft long plastic tube that was linked to a 1-mL syringe pre-filled with the medications.

Twenty rats were equally divided into four groups, including (a) the healthy rats as the healthy group, (b) the LPS-induced ALI rats with i.t. administration of saline as the LPS model group, (c) the LPS-induced ALI rats with i.t. administration of the JNEs reconstituted from JNDs by dissolving in saline (eq. to 0.5 mg JJBO) as the JNDs group, (d) the LPS-induced ALI rats with i.t. administration of Dexamethasone (DXM) Sodium Phosphate Injection (eq. to 0.5 mg DXM) as the DXM group. All the medicines were sprayed into the rat lungs through the trachea using a soft long plastic tube without anesthesia. The rats were deeply anesthetized with isoflurane 10 h post-administration. The right lungs were removed and the sections of three lobes were prepared for hematoxylin and eosin (H&E) staining, ELISA assay, and Western blotting, respectively.

H_2_O_2_-induced ALI was used in the study, which is the representative of chemical ALI [26]. Like the above process, 0.1 mL of 20 mM H_2_O_2_ was sprayed into the lungs through the trachea. Two hours later, therapy began with the same process as above. The rats were also divided to groups as the same as the above LPS experiment, where the H_2_O_2_-induced ALI rats were used to replace the LPS-induced ALI rats.

### 2.8. Histological Observation

The upper lobe of the right lungs was excised and fixed in a 10% formalin solution for at least 24 h, embedded in paraffin, and cut into 5-μm thick sections that were stained with H&E [27]. The pathological sections of lung tissues were observed under an optical microscope.

### 2.9. Caspase-3 Evaluation with Immunohistochemistry

The upper lobe of the right lungs was processed as mentioned above. The tissues were embedded in paraffin, deparaffined in xylene, and reconstituted with ethanol. The tissues were immersed in an ethylene diamine tetraacetic acid antigen retrieval solution (pH 8.0). The antigens were removed by microwave heating for 15 min. The sample was washed with water for 5 min and processed with an H_2_O_2_ solution (3%, 30 µL) to remove endogenous peroxidases. The primary antibodies of caspase-3 were added followed by incubation for 30 min at room temperature. The secondary antibodies (Goat Anti-Rabbit lgG-HRP) of the primary antibodies were used as above. Immunohistochemical detection was conducted. Stained sections were observed under a microscope.

### 2.10. Expression of NF-κB p65 with Western Blotting

NF-κB p65 in lung tissues was measured according to the report in [28]. The proteins in lung tissues were extracted and measured using the BCA kit. Moreover, NF-κB p65 proteins were separated with SDS-PAGE and then visualized after a series of immunohistochemical processes. The proteins were quantified using the Alpha software. All the reagents were obtained from the ServiceBio Technology Co., Ltd., Wuhan, China.

### 2.11. Measurement of Inflammatory and Oxidative Factors

The middle lobe of the right lungs was excised and weighed. Some pieces of tissues were 1:9 (v/v) mixed with ice-cold saline followed by thorough grinding in a high-speed tissue grinder (KI-II, ServiceBio Technology Co., Ltd., Wuhan, China). The homogenates were centrifuged at 4 °C, 12,000× *g* for 10 min and the supernatants were collected and preserved at −80 °C [29]. Measurements of TNF-α, IL-1β and IL-6, MDA, SOD, GSH and ROS were conducted with the commercially available corresponding ELISA assay kits and their contents were calculated according to the manufacturers’ instructions [2,30].

### 2.12. Assay of Elimination of Free Radical

JJBO’s function of eliminating free radicals can be evaluated with the scavenging activity of a stable free radical agent, DPPH. Electron spin resonance (ESR, A300, Bruker, Karlsruhe, Germany) was used to qualitatively evaluate the function of JNDs. The anti-free radical efficiency of JNDs was evaluated with UV-Vis spectrophotometry after DPPH reaction. A DPPH solution (0.13 mM, 3 mL) in ethanol was mixed with JJBO (1.5 mg), followed by storage for 30 min in the dark. JNDs (eq. 1.5 mg JJBO) were suspended in a DPPH solution (0.13 mM, 3 mL) in ethanol, followed by storage for 30 min in the dark. The mixtures and DPPH solution were separately measured using UV-Vis spectrophotometry at 517 nm. The anti-free radical efficiency of DPPH was the control. Anti-free radical efficiency was calculated as (A_control_ − A_sample_)/A_control_ ×100%.

### 2.13. Statistical Analysis

All the results were expressed as means ± standard deviations (SD). A one-way ANOVA test was performed for all statistical evaluations. Individual differences between groups were identified using Dunn’s test. Statistical significance was defined as *p* < 0.05 or <0.01.

## 3. Results

### 3.1. Characteristics of JNEs and JNDs

**Figure 1:**
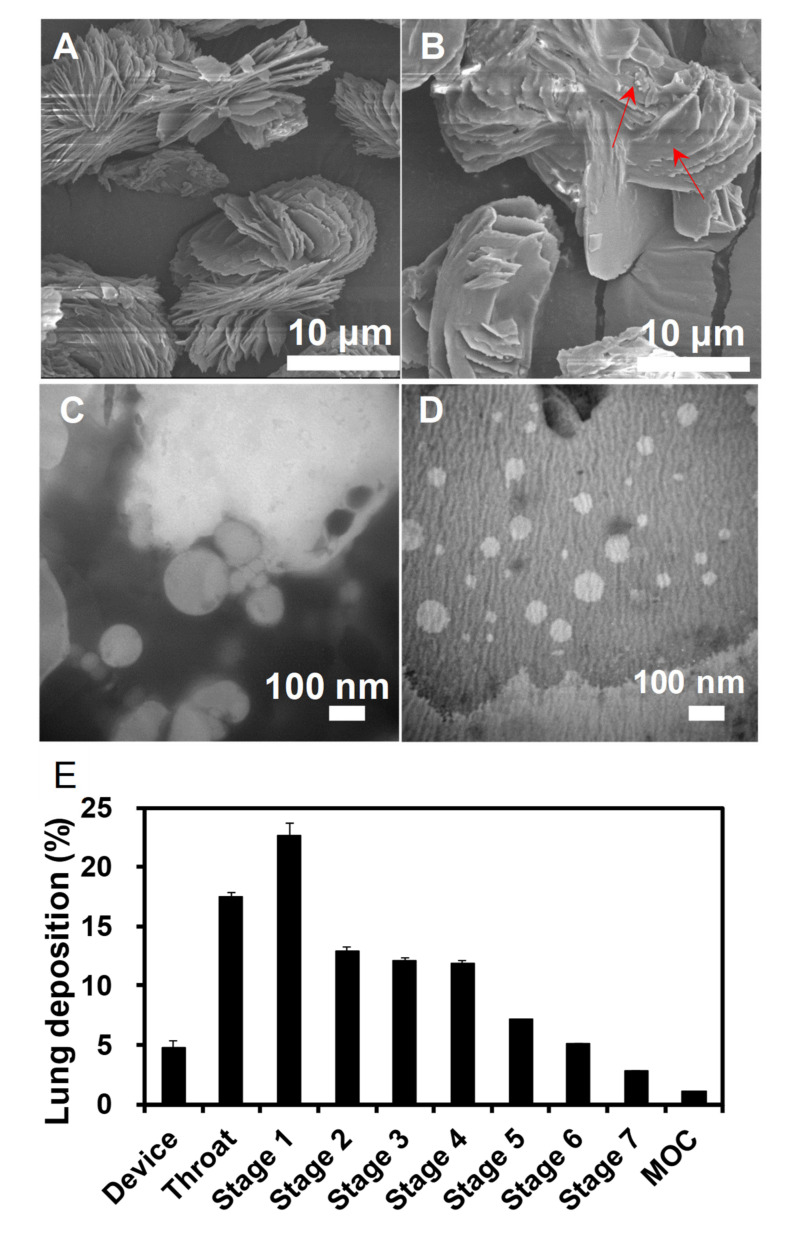
SEM images of blank excipients (**A**) and jojoba oil (JJBO) dry nanoemulsion powders (JNDs) (**B**). TEM images of the original JJBO nanoemulsions (JNEs) (**C**) and the JNEs reconstituted from JNDs (**D**). JJBO in the JNDs depositing in the Next Generation Impactor (NGI) (**E**). The data are presented as means ± SD (*n* = 3). The arrows indicate the JNE particles.

JNEs were white oil-in-water (O/W) emulsions with the mean size of 71.97 ± 6.57 nm (*n* = 3), the polydispersity index (PDI) of 0.25 ± 0.01 (*n* = 3) and the zeta potential of −50.43 ± 1.79 mV (*n* = 3). JNEs were stable due to their high surface charge. No precipitates appeared in the emulsions 12 h post-preparation. JNDs were white loose powders. Figure 1A shows the state of the lyophilized excipients except for JJBO, involving lecithin and lactose. The particles in Figure 1A were smooth but some were tiny particles adsorbed on the surface of the large particles in Figure 1B. The tiny particles should be the lyophilized JNEs. JNDs had a relatively high *D_e_* of 12.65 ± 0.15 µm (*n* = 3) but a very low tap density of 0.170 ± 0.010 g/cm^3^ so that the *D_a_* was only 5.86 ± 0.13 µm (*n* = 3). Moreover, the MMAD of JNDs was 4.17 µm and the FPF was 39.11%, benefiting lung inhalation (Figure 1E). Therefore, JNDs were suitable for deposition in the deep sites of the lung after inhalation. More importantly, JNEs were quickly reconstituted from JNDs within 1 min, which had similar spherical morphology and sizes (~70 nm) to the original JNEs (Figure 1C,D). Therefore, JNEs would also quickly be reconstituted after pulmonary delivery of JNDs.

### 3.2. JNDs Attenuate LPS-Induced ALI

**Figure 2:**
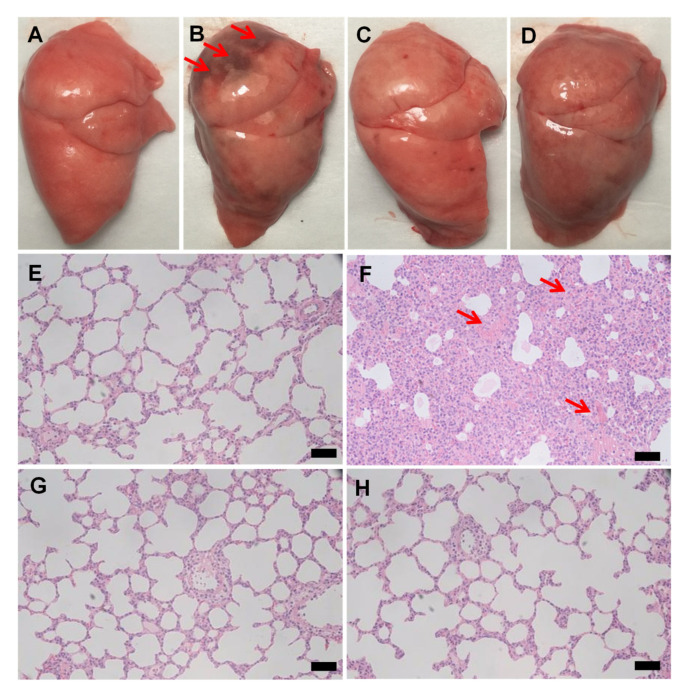
The appearance of the lung tissues from the healthy rats (**A**), the lipopolysaccharide (LPS)-induced model group (**B**), the dexamethasone (DXM) group (**C**), and the JNDs group (**D**). Hematoxylin and eosin (H&E) images of the lung tissues from the healthy rats (**E**), the LPS-induced model group (**F**), the DXM group (**G**), and the JNDs group (**H**). The scale bars indicate 100 µm. The red arrows indicate lung injuries and significant hemorrhage sites.

LPS was a severe lung injury factor, which was significantly exhibited in the model group (Figure 2A,B). The JNDs and DXM groups also showed lung injuries with hemorrhage but much less than the model group (Figure 2C,D); furthermore, they showed few inflammatory cells in the lung tissues compared to the obvious infiltration of monocytes and neutrophils of the model groups (Figure 2E–H). Therefore, JNDs have high anti-LPS-induced ALI effect.

**Figure 3:**
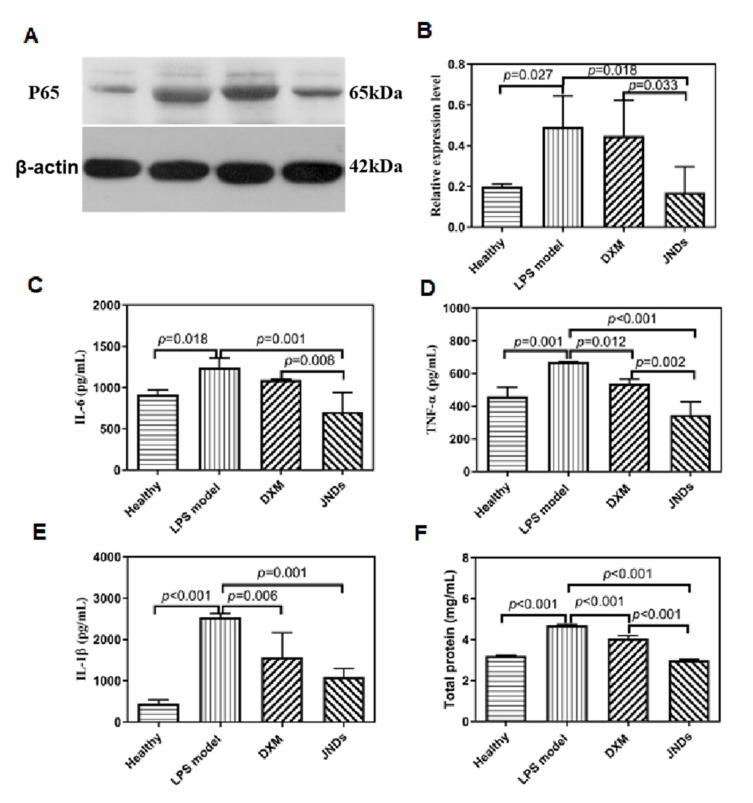
(**A**) Western blotting images of NF-κB p65 proteins. Left to right: the healthy rats, the LPS-induced ALI rats, the DXM group, and the JNDs group. (**B**) Relative expression levels of NF-κB p65 proteins compared to the actin standards in different groups according to Graph (**A**); (**C**) expressions of IL-6 according to Graph (**A**); (**D**) expressions of TNF-α according to Graph (**A**); (**E**) expressions of IL-1β according to Graph (**A**); (**F**) total proteins in the lung tissues from the different groups. The data are presented as means ± SD (*n* = 3).

The excessive activation of NF-κB p65 was observed in the LPS-induced ALI rat compared to that in the healthy rats. The DXM group showed a similar expression level of NF-κB p65 to the model group while JNDs significantly inhibited the expression of NF-κB p65 in the lung tissues (Figure 3A,B). Therefore, JNDs have the anti-inflammatory function by inhibiting the NF-κB p65 pathway.

The expressions of IL-6, IL-1β and TNF-α in the JNDs group were also significantly lower than those in the model group. Although some expressions in the DXM group also showed the statistical differences compared with the same factor levels in the model group, almost all the values were obviously higher than those in the JNDs group; furthermore, some expressions had the statistical differences between the two treatment groups (Figure 3C–F). These results were almost consistent with the expression profiles of NF-κB p65. Severe inflammation would likely damage the endothelial and epithelial systems, facilitating the influx of protein-rich fluids and then leading to pulmonary edema [31]. The number of total proteins from the JNDs group was the lowest with very significant differences compared to those from the model and DXM groups.

### 3.3. JNDs Attenuate H_2_O_2_-Induced ALI

**Figure 4:**
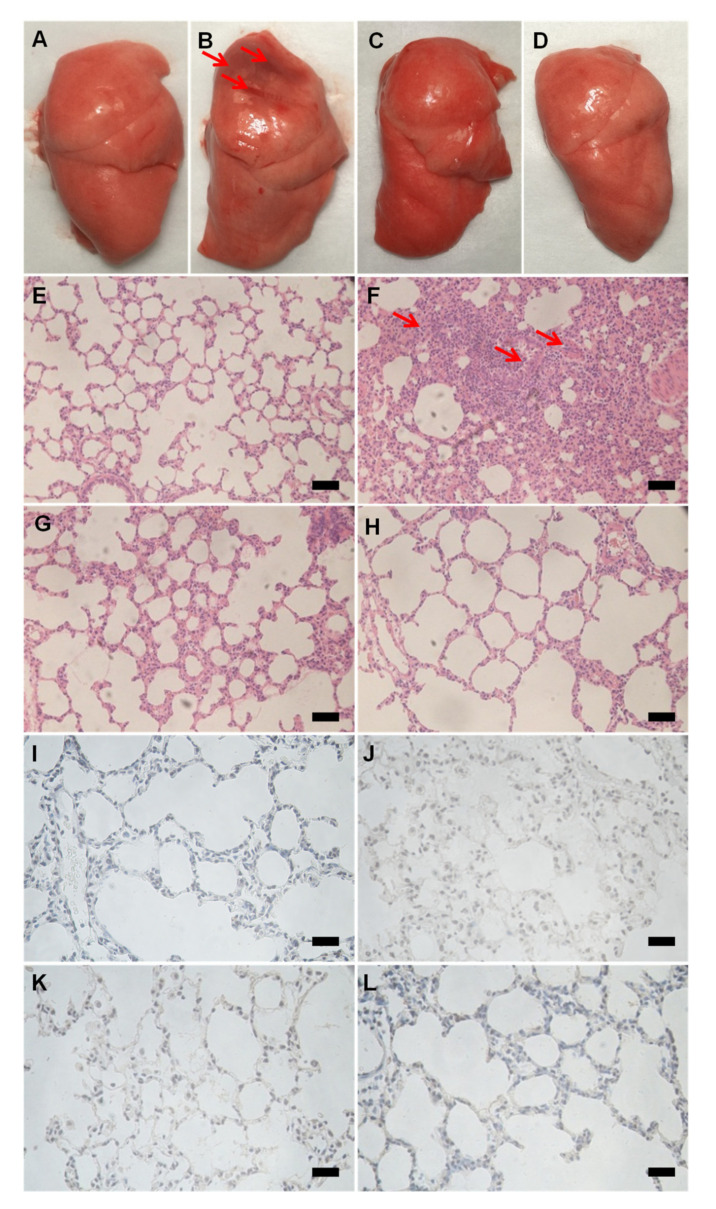
Appearance, H&E images and caspase-3 staining of the lung tissues from the healthy rats (**A**,**E**,**I**), the H_2_O_2_-induced ALI rats (**B**,**F**,**J**), the DXM group (**C**,**G**,**K**), and the JNDs group (**D**,**H**,**L**). The scale bars indicate 100 µm.

Like the LPS experiments, JNDs also showed a remarkable anti-H_2_O_2_-induced ALI effect. The i.t. administration of H_2_O_2_ also induced severe hemorrhage in the lung tissues (Figure 4). Both the treatments of JNDs and DXM resulted in little bleeding in the lung tissues. Pathological sections further showed the obvious hemorrhage and infiltration of monocytes and neutrophils in the H_2_O_2_-induced ALI rat lungs (Figure 4F), while JNDs and DXM remarkably attenuated the lung injury.

H_2_O_2_-induced ALI affects apoptosis [32]. In this study, the high caspase-3 expression appeared in the lung tissue sections from the H_2_O_2_-induced ALI rats compared to the healthy rats (Figure 4I,J), while the i.t. administration of JNDs and DXM remarkably attenuated caspase-3 expression (Figure 4K,L).

**Figure 5:**
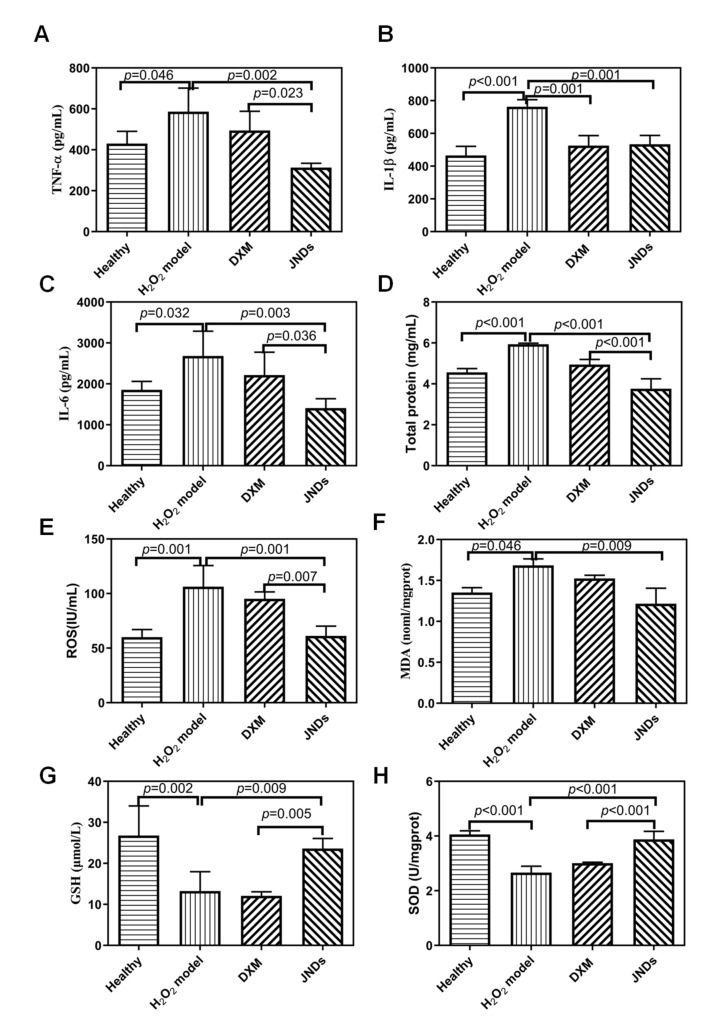
Expressions of TNF-α (**A**), IL-1β (**B**), IL-6 (**C**), total proteins (**D**), reactive oxygen species (ROS) (**E**), malondialdehyde (MDA) (**F**), glutathione (GSH) (**G**) and superoxide dismutase (SOD) (**H**). The data are presented as means ± SD (*n* = 3).

Like LPS experiments, some important inflammatory factors were also measured. JNDs remarkably deregulated the levels of pro-inflammatory mediators (TNF-α, IL-1β, and IL-6) compared to the H_2_O_2_-induced ALI model (Figure 5A–C), indicating that JNDs had the strong anti-inflammation function. Moreover, the effects of JNDs were statistically different from those of DXM (*p* < 0.05 or *p* < 0.01). The number of total proteins in the JNDs group was also obviously lower than those in the model and DXM groups, indicating that JNDs suppressed the H_2_O_2_-induced lung edema (Figure 5D). Therefore, JNDs have strong anti-H_2_O_2_-induced ALI function.

**Figure 6:**
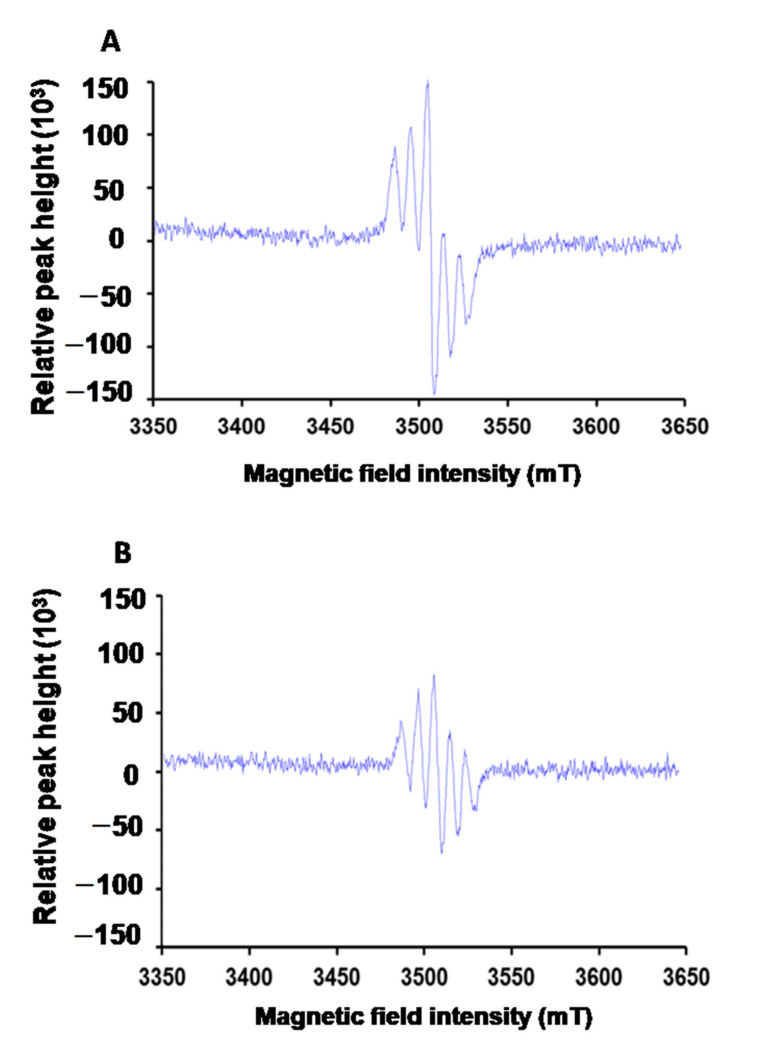
Anti-free radical efficiency of DPPH (**A**) and JNDs (**B**).

JNDs also had strong free radical scavenging function, which was confirmed with the in vitro experiments (Figure 6). The anti-free radical efficiencies of JJBO and JNDs were 29.89% and 31.57%, respectively. It may be an important mechanism of JND function. The in vivo studies further demonstrated that JNDs had the significant free radical scavenging function with the much lower levels of ROS and MDA in the lung tissues than those from the H_2_O_2_-induced ALI model and DXM groups (Figure 5). Moreover, the reduction factors, including GSH and SOD, were much higher in the JNDs group than those in the ALI model and DXM groups, whereas the DXM group had similar oxidation and reduction levels to the ALI model. Therefore, JNDs effectively attenuate the oxidative stress for the treatment of H_2_O_2_-induced ALI.

## 4. Discussion

JJBO is a light-yellow oil and is mainly composed of linear long-chain esters. It has many medicinal applications such as anti-inflammation [7], wound healing, skin disorder healing [6], and antioxidant [33]. However, the potential of JJBO as a highly anti-inflammatory natural plant oil is unlikely to be fully realized due to its disadvantage of low solubility and poor dispensability. Here, JNEs as a stable delivery system were obtained, which made JJBO uniformly distributed in the dry powders after lyophilization. JNDs with the appropriate MMAD and FPF were suitable for deposition in the deep sites of the lung. More importantly, JNEs were rapidly reconstituted from JNDs. Therefore, JJBO could be well dispersed in lung tissues after JNDs were i.t. administered, which benefited the topical effectiveness of drugs.

LPS, as a component of the cell wall of Gram-negative bacteria, is an endotoxin. Here, we verified that LPS induced significant hemorrhage and obvious infiltration of monocytes and neutrophils in the lung tissues. JNDs highly attenuated the bleeding degree and the inflammatory cell infiltration. When LPS were inhaled into the lungs, LPS-binding proteins are formed and then transported to the surface of immune cell membranes to promote the binding of LPS to the membrane surface protein CD14. Subsequently, CD14 further transports LPS to the Toll-like receptor 4 to trigger a cascade of responses that activate the transcription factor NF-κB [34,35]. Therefore, the NF-κB transcription factor is considered as a central regulator of inflammation. Here, JNDs remarkably inhibited the expression of NF-κB p65 of the lung tissues, which demonstrated JNDs could have high anti-inflammatory activity by regulating the NF-κB pathway. NF-κB is a regulatory point of the inflammatory response [36]. LPS can activate the NF-κB pathway and then induce the production of neutrophil inflammatory cytokines (i.e., IL-6, IL-1β, and TNF-α,) [37]. Moreover, IL-6, IL-1β and TNF-α are key cytokines that trigger inflammatory responses and accurately reflect NF-κB-mediated inflammatory levels [38]. JNDs significantly decreased the expression levels of IL-6, IL-1β, and TNF-α and the number of total proteins in the LPS-induced ALI lung tissues. Therefore, JNDs alleviated the LPS-induced damage in the lung tissues by inhibiting the NF-κB mediated inflammatory pathways.

H_2_O_2_ has strong oxidizing effect that easily damages lung tissues. We verified that H_2_O_2_ not only induced hemorrhage and the infiltration of inflammatory cells but also promoted cell apoptosis in lung tissues. Gallic acid, an endogenous plant phenol, is one of the main ingredients of JJBO, which has excellent oxidation resistance and inhibits caspase-3 activation [39]. Therefore, JNDs as one type of inhalable powders for JJBO highly reduced oxidative stress induced by H_2_O_2_ and played a protective role in the lung tissues. JNDs had strong anti-inflammatory activity as confirmed by the low levels of pro-inflammatory mediators and few total proteins in lung tissues. The positive anti-inflammatory effect of JNDs on H_2_O_2_-induced ALI partly resulted from their high capacity for free radical scavenging. It is known that free radicals elevate the ROS and MDA content and decrease the SOD activity and the GSH content. In contrast, DXM was far less effective than JNDs on the treatment of H_2_O_2_-induced ALI without free radical scavenging.

The caspase family is a group of cysteine proteases that play an important role in apoptosis. Caspase-3 is considered to be a key protein for cell apoptosis [39]. After activation, it inhibits the protein, extracellular matrix and matrix protein, DNA transcription, replication and repair related proteins through enzymatic hydrolysis of apoptosis, resulting in cell condensation, separation from surrounding cells, chromatin aggregation, etc., and finally leading to the irreversible apoptosis of cells [40]. JNDs effectively attenuated the expression of caspase-3 improved by H_2_O_2_-induced ALI so that a series of apoptosis processes were hindered.

Oxygen free radicals are produced after inhalation of H_2_O_2_ with large quantities, exceeding the body’s ability to scavenge, and then do damage to the pulmonary vascular endothelial cells/alveolar epithelial cells, leading to ALI [41]. JJBO has good antioxidant capacity due to its highly radical scavenging activity [33]. Here, we confirm the capacity of JJBO in the form of inhalable JNDs for the scavenging of free radicals and the treatment of ALI.

## 5. Conclusions

JJBO is mainly composed of linear long-chain esters and grows in desert and semi-desert areas. It has high power for the regulation of the NF-κB p65 pathway and radical scavenging. However, the potential of JJBO as a highly anti-inflammatory natural plant oil is unlikely to be fully realized due to its disadvantage of low solubility and poor dispersibility. We used inhalable JNDs and the pulmonary delivery method to solve the application challenges of JJBO as an effective medication for the treatment of ALI, a common lung disease. Not only was an optimal formulation of inhalable JNDs obtained, but also the anti-LPS- and H_2_O_2_-induced ALI mechanisms were also well explored. The new dosage form and pulmonary delivery method improved the JJBO wider applications more than before. Inhalable JNDs are a promising anti-inflammatory and anti-free radical medication for the treatment of ALI. We hope that JJBO and its formulations with high safety and ideal treatment for ALI can provide help for the new coronavirus pneumonia (COVID-19).

## Data Availability

Not applicable.

## References

[1] El-Mallah M.H., El-Shami S.M. (2009). Investigation of liquid wax components of Egyptian jojoba seeds. J. Oleo Sci..

[2] Elshazly M.O., Morgan A.M., Ali M.E., Abdel-mawla E., El-Rahman S.S.A. (2016). The mitigative effect of Raphanus sativus oil on chromium-induced geno-and hepatotoxicity in male rats. J. Adv. Res..

[3] Galati G., O’brien P.J. (2004). Potential toxicity of flavonoids and other dietary phenolics: Significance for their chemopreventive and anticancer properties. Free Radic Biol. Med..

[4] Servili M., Esposto S., Fabiani R., Urbani S., Taticchi A., Mariucci F., Selvaggini R., Montedoro G.F. (2009). Phenolic compounds in olive oil: Antioxidant, health and organoleptic activities according to their chemical structure. Inflammopharmacology.

[5] Ranzato E., Martinotti S., Burlando B. (2011). Wound healing properties of jojoba liquid wax: An in vitro study. J. Ethnopharmacol..

[6] Lin T.-K., Zhong L., Santiago J.L. (2018). Anti-inflammatory and skin barrier repair effects of topical application of some plant oils. Int. J. Mol. Sci..

[7] Habashy R.R., Abdel-Naim A.B., Khalifa A.E., Al-Azizi M.M. (2005). Anti-inflammatory effects of jojoba liquid wax in experimental models. Pharmacol. Res..

[8] Ibrahim H.M., Abou-Arab A.A., Salem F.M.A. (2011). Antioxidant and antimicrobial effect of some natural plant extracts added to lamb patties during storage. Grasas Y Aceites.

[9] Loy H., Kuok D.I., Hui K.P., Choi M.H., Yuen W., Nicholls J.M., Peiris J.M., Chan M.C. (2018). Therapeutic implications of human umbilical cord mesenchymal stromal cells in attenuating influenza A/H5N1-associated acute lung injury. J. Infect. Dis..

[10] Zhang Y., Lv R., Hu X., Jiang L., Xiao D., Sun Y., Zhao J., Bao Q., Xie J. (2017). The role of IL-33 on LPS-induced acute lung injury in mice. Inflammation.

[11] Wheeler A.P., Bernard G.R. (2007). Acute lung injury and the acute respiratory distress syndrome: A clinical review. Lancet.

[12] Whitea A.F.B., Demchenko A.V. (2014). Modulating LPS signal transduction at the LPS receptor complex with synthetic Lipid A analogues. Adv. Carbohydr. Chem. Biochem..

[13] Kundu M., Sadhukhan P., Ghosh N., Chatterjee S., Manna P., Das J., Sil P.C. (2019). pH-responsive and targeted delivery of curcumin via phenylboronic acid-functionalized ZnO nanoparticles for breast cancer therapy. J. Adv. Res..

[14] Schreiber M.P., Colantuoni E., Bienvenu O.J., Neufeld K.J., Chen K.-F., Shanholtz C., Mendez-Tellez P.A., Needham D.M. (2014). Corticosteroids and transition to delirium in patients with acute lung injury. Crit. Care Med..

[15] Yang M.Y., Chan J.G.Y., Chan H.-K. (2014). Pulmonary drug delivery by powder aerosols. J. Control. Release.

[16] Wang J., Li X., Liu M., Wang S., Cao Z. (2016). Inhibitory effect of Zanthoxylum bungeanum seed oil on ovalbumin-induced lung inflammation in a murine model of asthma. Mol. Med. Rep..

[17] Tu G., Shi Y., Zheng Y., Ju M., He H., Ma G., Hao G., Luo Z. (2017). Glucocorticoid attenuates acute lung injury through induction of type 2 macrophage. J. Transl. Med..

[18] Perlman S. (2019). Another Decade, Another Coronavirus. N. Engl. J. Med..

[19] Silvaa D.M., Palecob R., Trainib D., Sencadas V. (2018). Development of ciprofloxacin-loaded poly(vinyl alcohol) dry powder formulations for lung delivery. Int. J. Pharm..

[20] Zhu L., Li M., Dong J., Jin Y. (2015). Dimethyl silicone dry nanoemulsion inhalations: Formulation study and anti-acute lung injury effect. Int. J. Pharm..

[21] Cheng S., Kourmatzis A., Mekonnen T., Gholizadeh H., Raco J., Chen L., Tang P., Chan H.-K. (2019). Does upper airway deformation affect drug deposition?. Int. J. Pharm..

[22] Hu Y., Li M., Zhang M., Jin Y. (2018). Inhalation treatment of idiopathic pulmonary fibrosis with curcumin large porous microparticles. Int. J. Pharm..

[23] Lercker G., Rodriguez-Estrada M.T. (2000). Chromatographic analysis of unsaponifiable compounds of olive oils and fat-containing foods. J. Chromatogr. A.

[24] Dewanto V., Wu X., Adom K.K., Liu R. (2002). Thermal processing enhances the nutritional value of tomatoes by increasing total antioxidant activity. J. Agric. Food Chem..

[25] Wan L., Tan J., Wan S., Meng D., Yu P. (2016). Anti-inflammatory and anti-oxidative effects of dexpanthenol on lipopolysaccharide induced acute lung injury in mice. Inflammation.

[26] Belhadj S., Hentati O., Hamdaoui G., Fakhreddine K., Maillard E., Dal S., Sigrist S. (2018). Beneficial effect of jojoba seed extracts on hyperglycemia-Induced oxidative stress in RINm5f beta cells. Nutrients.

[27] Li M., Zhu L., Liu B., Du L., Jia X., Han L., Jin Y. (2016). Tea tree oil nanoemulsions for inhalation therapies of bacterial and fungal pneumonia. Colloids Surf. B Biointerfaces.

[28] Fernández D.J., Lamkanfi M. (2015). Inflammatory caspases: Key regulators of inflammation and cell death. Biol. Chem..

[29] Sani D., Khatab N.I.O., Kirby B.P., Yong A., Hasan S., Basri H., Stanslas J. (2018). A standardised Andrographis paniculata Burm. Nees aqueous extract prevents Lipopolysaccharide-induced cognitive deficits through suppression of inflammatory cytokines and oxidative stress mediators. J. Adv. Res..

[30] Bhatia M., Moochhala S. (2004). Role of inflammatory mediators in the pathophysiology of acute respiratory distress syndrome. J. Pathol..

[31] Jiao C., Chen W., Tan X., Liang H., Li J., Yun H., He C., Chen J., Ma X., Xie Y. (2020). Ganoderma lucidum spore oil induces apoptosis of breast cancer cells in vitro and in vivo by activating caspase-3 and caspase-9. J. Ethnopharmacol..

[32] Zhao H., Zeng Z., Liu L., Chen J., Zhou H., Huang L., Huang J., Xu H., Xu Y., Chen Z. (2018). Polydopamine nanoparticles for treatment of acute inflammationinduced injury. Nanoscale.

[33] Badr A.N., Shehata M.G., Abdel-Razek A.G. (2017). Antioxidant activities and potential impacts to reduce aflatoxins utilizing jojoba and jatropha oils and extracts. Int. J. Pharmacol..

[34] Pło´ciennikowska A., Hromada-Judycka A., Borzecka K., Kwiatkowska K. (2015). Co-operation of TLR4 and raft proteins in LPS-induced pro-inflammatory signaling. Cell Mol. Life Sci..

[35] Lu Y., Yeh W., Ohashi P.S. (2008). LPS/TLR4 signal transduction pathway. Cytokine.

[36] Schwarz A., Bonaterra G.A., Schwarzbach H., Kinscherf R. (2017). Oxidized LDL-induced JAB1 influences NF-kappaB independent inflammatory signaling in human macrophages during foam cell formation. J. Biomed. Sci..

[37] Yang H., Zhuo J., Sun C., Nie J., Yuan J., Liu Y., Lin R., Lai X., Sua Z., Li Y. (2018). Pogostone attenuates TNF-alpha-induced injury in A549 cells via inhibiting NF-kappaB and activating Nrf2 pathways. Int. Immunopharmacol..

[38] Shojaie M., Ghanbari F., Shojaie N. (2017). Intermittent fasting could ameliorate cognitive function against distress by regulation of inflammatory response pathway. J. Adv. Res..

[39] Shalini S., Dorstyn L., Dawar S., Kumar S. (2015). Old, new and emerging functions of caspases. Cell Death Differ..

[40] Pizzino G., Irrera N., Cucinotta M., Pallio G., Mannino F., Arcoraci V., Squadrito F., Altavilla D., Bitto A. (2017). Oxidative stress: Harms and benefits for human health. Oxid. Med. Cell Longev..

[41] Abdel-Mageed W.M., Bayoumi S.A.L.H., Salama A.A.R., Salem-Bekhit M.M., Abd-Alrahman S.H., Sayed H.M. (2014). Antioxidant lipoxygenase inhibitors from the leaf extracts of Simmondsia chinensis. Asian Pac. J. Trop. Med..

